# Supplementary material for: Ferromagnetic soft catheter robots for minimally invasive bioprinting
Source: Nat Commun. 2021 Aug 20;12:5072. doi: 10.1038/s41467-021-25386-w (PMC8379157; doi:10.1038/s41467-021-25386-w)
Supplement: Supplementary file 1 — Supplementary Information [file 41467_2021_25386_MOESM1_ESM.pdf]

Supplementary Information

Ferromagnetic Soft Catheter Robots for Minimally Invasive Bioprinting

Cheng Zhou[1,6], Youzhou Yang[1,6], Jiaxin Wang[2], Qingyang Wu[1], Zhuozhi Gu[1], Yuting Zhou[3], Xurui Liu[1], Yueying Yang[1], Hanchuan Tang[1], Qing Ling[2], Liu Wang[4]†, Jianfeng Zang[1,5]†

1. School of Optical and Electronic Information and Wuhan National Laboratory for Optoelectronics, Huazhong University of Science and Technology, Wuhan 430074, China

2. Department of Urology, Tongji Hospital, Tongji Medical College, Huazhong University of Science and Technology, Wuhan 430064, China

3. The Key Laboratory of Bionic Engineering (Ministry of Education) and the College of Biological and Agricultural Engineering, Jilin University, 5988 Renmin Street, Changchun 130025, China

4. CAS Key Laboratory of Mechanical Behavior and Design of Materials, Department of Modern Mechanics, University of Science and Technology of China, Hefei, Anhui 230026, China

5. The State Key Laboratory of Digital Manufacturing Equipment and Technology, Huazhong University of Science and Technology, Wuhan 430074, China

6. These authors contributed equally: Cheng Zhou, Youzhou Yang

†Corresponding author. Email: jfzang@hust.edu.cn; liuwang@mit.edu

Supplementary Notes

Supplementary Note 1: Pressure loss calculation for viscoelastic inks

As can be seen from Supplementary Fig. 10 below, the apparent viscosity decreases with increasing shear rate, indicating that the silicone inks are non-Newtonian fluids and exhibit shear thinning characteristics. Flow characteristics can be described in modified Herschel-Bulkley model where the apparent viscosity is given by:

$$\tau = \tau_y + j(\gamma)^n \tag{1}$$

We consider ink printing to be laminar rather than turbulent flow so the result for the pressure loss is:

$$\frac{\Delta P}{L} = \frac{4j}{D}(\frac{8V}{D})^n(\frac{3n+1}{4n})^n(\frac{1}{1-X})(\frac{1}{1-aX-bX^2-cX^3})^n \tag{2}$$

and X is given by

$$X = \frac{4L\tau_y}{D\Delta P} \tag{3}$$

and,

$$a = \frac{1}{(2n+1)} \tag{4}$$

$$b = \frac{2n}{(n+1)(2n+1)} \tag{5}$$

$$c = \frac{2n^2}{(n+1)(2n+1)} \tag{6}$$

where n is Herschel-Bulkley index and j is Herschel-Bulkley parameters for a particular fluid, τ_y is yield stress, ΔP is pressure loss, L is pipe length, V is mean pipe velocity and D is pipe diameter.

In our experiment, j , n , τ_y can be obtained from a curve-fit to the rheological data in Supplementary Fig. 10c. Fitting the shear stress and shear rate of printing ink (PDMS : Ecoflex 1:1), we obtained j = 37.16, n = 0.7196, τ_y = 336.2 N/m^2 , which shows that the material is a non-Newtonian fluid with initial shear yield stress and shear thinning characteristics because of $j > 0$, $n < 1$. Other parameters are measured and we calculate the pressure loss ΔP using the data above (Supplementary Fig. 4). All parameters are listed in **Supplementary Table 3**.

Supplementary Note 2: Cylindrical coordinate moving system

As Supplementary Fig. 8a shows, the distribution of the magnetic field is non-uniform. For example, in XY plane, radials of different angle has different magnetic distribution, especially in the angle of 0 ° and 45 °, which were verified by both analytical calculation and experimental measurements (Supplementary Fig. 8a). Thus, if we choose a Cartesian coordinate system to move the motor-driven magnetic field, the FSCR will be driven by the whole field at every angle making it more difficult to establish the tip-field mapping relationship. However, a cylindrical moving coordinate system will only use the magnetic field at the angle of 0 °. Moreover, owing to the fringe field effect, the strength and direction of the magnetic field will change undesirably in diagonal plane (angle 45 °). However, we only need to map the tip-field relationship on translational axis r (angle 0 °) for a cylindrical coordinate system. Consequently, a cylindrical moving coordinate system not only take full advantage of the maximum strength and gradient of the field, but also improves the stability and linearity of the control.

Supplementary Note 3: Conductivity testing of hydrogel ink

The two probe testing method was used to test the conductivity of the conducting hydrogel as previous reported. The gap L between the two glass carbon electrodes, the inner diameter D of cylindrical mold and the diameter of electrode d were 3 mm, 6 mm and 3 mm, respectively. The impedance of the conducting hydrogels was recorded at 5 mV over a range of frequencies from 10^{-2} to 10^{5} Hz. The conductivity was calculated using Supplementary Equation 7:

$$\sigma = \frac{\mathrm{Re}(Z)}{[\mathrm{Re}(Z)]^2 + [\mathrm{Im}(Z)]^2} * \frac{L}{S} \tag{7}$$

where $\mathrm{Re}(Z)$ and $\mathrm{Im}(Z)$ are the real and imaginary parts of the complex impedance of the sample at 1Hz and S is area of the electrode. Thus, the conductivity is ~10 S/m.

Supplementary Tables

Supplementary Table 1. Summary of various mechanisms for in vivo bioprinting

Type	Printing depth	Incision size	Printing area	Compliance of nozzle	Material delivery
Conventional method[11,12]	Surface (Epidermal)	Traditional surgery	-	Rigid	Direct injection
Near-infrared polymerization [13,14]	<10 mm (Subcutaneous Tissue)	Non-invasive-	<0.2 cm^2	-	Pre-injection and need container
Micromachine printer[16]	>100 mm (Inside body)	~28 mm (minimally invasive)	~ 9 cm^2	Rigid	Direct injection
**FSCR printing system**	**0 - 150 mm (Inside body)**	**2~4 mm (minimally invasive)**	**0~27 cm^2**	**Deformable (~1.15 MPa)**	**Direct injection**

Supplementary Table 2. Comparison between our FSCR and representative commercially-available catheters

Design/Model	Functionality	Control mechanism	Control apparatus	Reference
Climber (Terumo, Europe)	Guide	Manual pushing/ twisting	Hand	Terumo Europe product catalog
Navistar (Biosense Webster, USA)	Navigation	Magnetic/manual	CARTO® 3 System (Complex operating system)	Biosense Webster product catalog
ACUSON (AcuNav, USA)	Ultrasound catheter	Magnetic/manual	V-Sono™ ICE catheter manipulator and Genesis RMN System (Complex operating system)	ACUSON product catalog
FSCR	Bioprinting	Magnetic/automated	Four magnets (Simple)	Our work

Supplementary Table 3. Parameters measured for estimating pressure loss

$P_{set}(MPa)$	j	n	$\tau_y(N/m^2)$	$L(m)$	$V(m/s)$	$D(m)$	$\Delta P(MPa)$
0.135	37.16	0.7196	336.6	0.09	8.2e-4	0.0013	0.128
0.170	37.16	0.7196	336.6	0.09	9.7e-4	0.0013	0.132
0.205	37.16	0.7196	336.6	0.09	1.16e-3	0.0013	0.136
0.240	37.16	0.7196	336.6	0.09	1.28e-3	0.0013	0.139
0.275	37.16	0.7196	336.6	0.09	1.49e-3	0.0013	0.143

Supplementary Figures

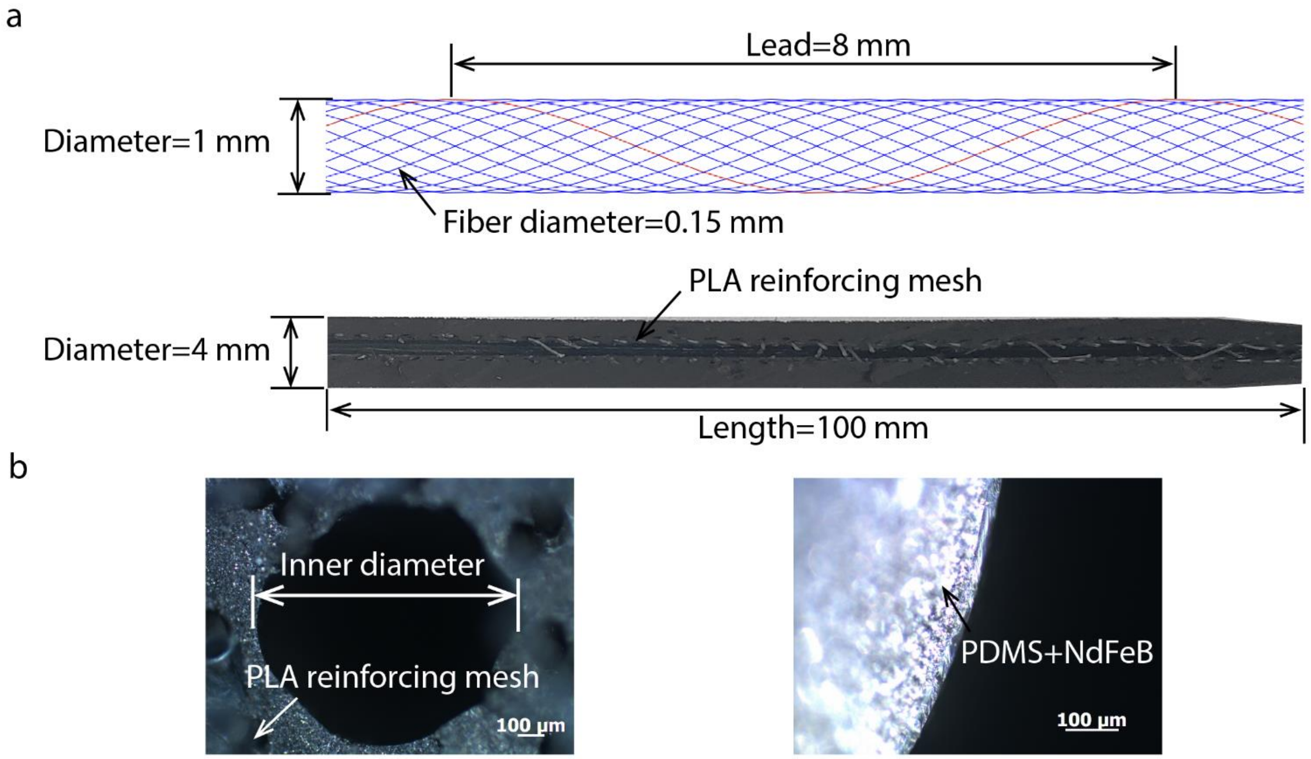

Supplementary Figure 1. The design parameters of PLA-fiber reinforced FSCR. (a) PLA reinforcing mesh and longitudinal section of a FSCR. **(b)** Optical microscope images of a FSCR's cross section. A large number of FSCRs are fabricated, and the molding method of FSCR make the structure parameters reproducibility.

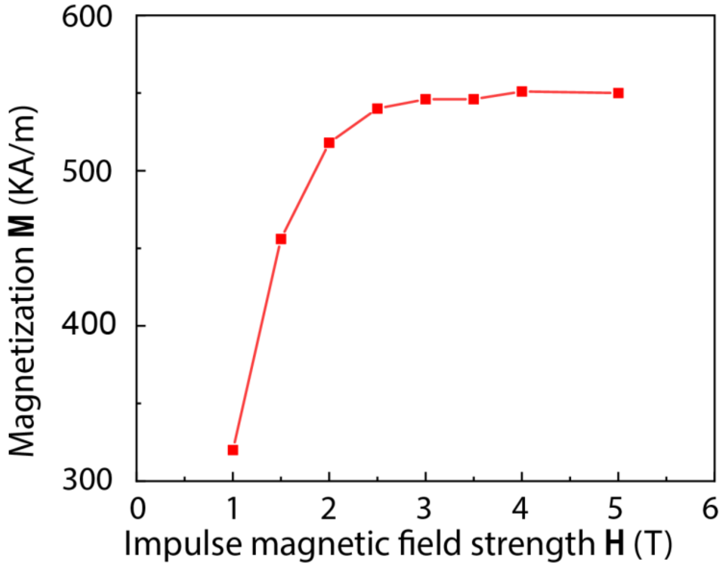

Supplementary Figure 2. Residual Magnetization of FSCR. Dependence of the magnitude of residual magnetization, **M**, of NdFeB magnetic particles on the strength, **H**, of impulse magnetic field. Saturation occurs when the magnetic field strength reaches ~3T.

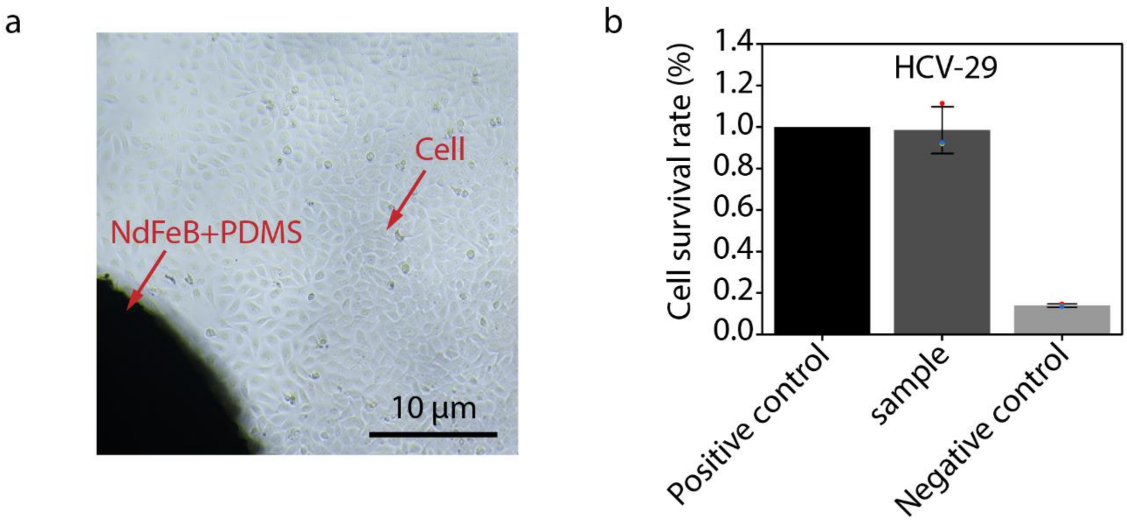

Supplementary Figure 3. **Biocompatibility tests of the cured NdFeB +PDMS composite.** (a) Images of HCV-29 cells cultured with cured PDMS+NdFeB composite after 24 h. (b) The cell survival rate is 98.6%, suggesting high biocompatibility of our FSCR according to the standard (70% cell survival rate) of USP (ISO 10993-5). Error bars represent Standard Deviation (n=3).

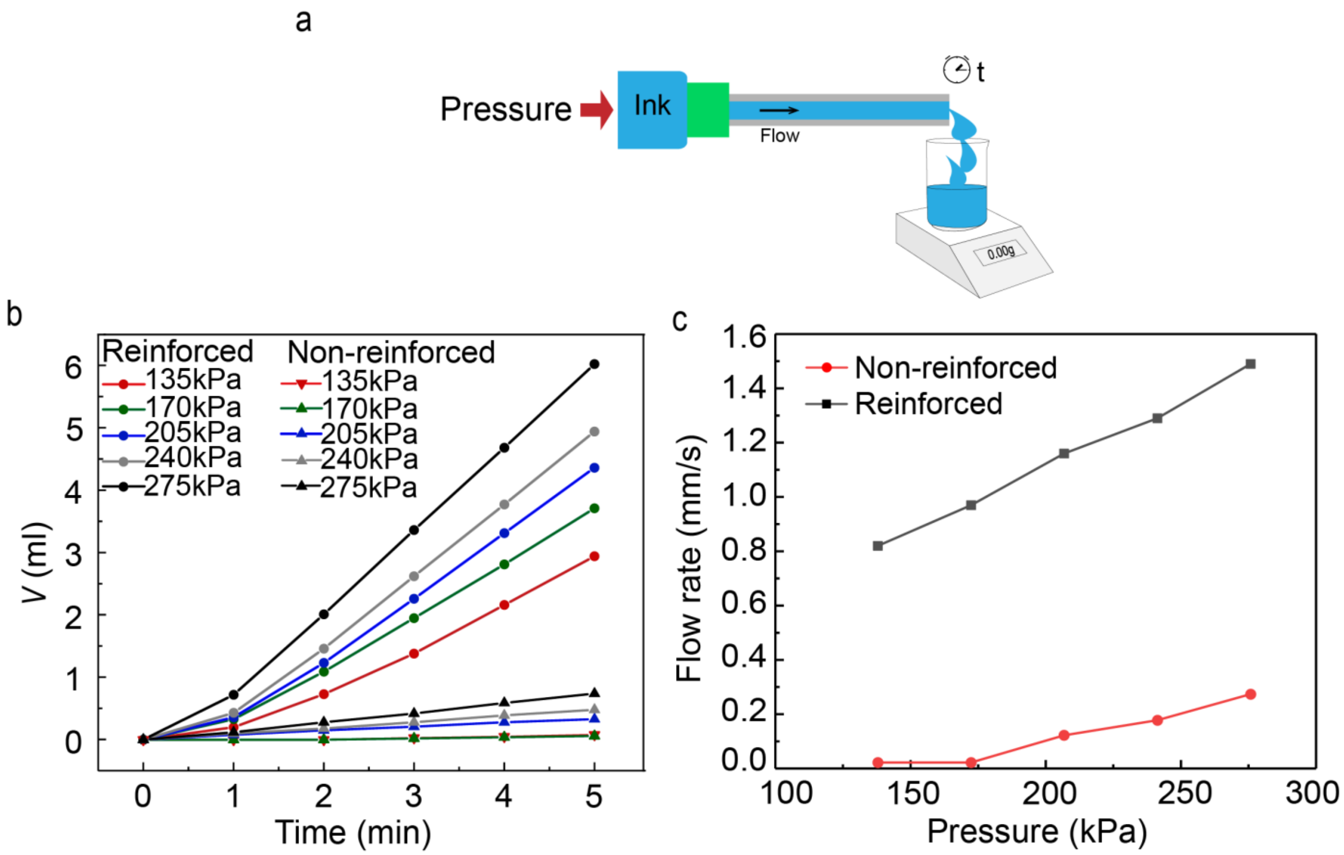

Supplementary Figure 4. Comparison of flow and flow rate of the reinforced and non-reinforced catheter robots. (a) Illustration of measuring critical parameter flow for calculating inner pressure with variable applied pressure. **(b)** A variable applied pressure source is placed in series with an ink-filled (ink: the weight ratio of SE 1700 to Ecoflex part-A is 1:1) catheter robot to measure flow over time. **(c)** Plot of flow rate versus pressure under steady flow conditions.

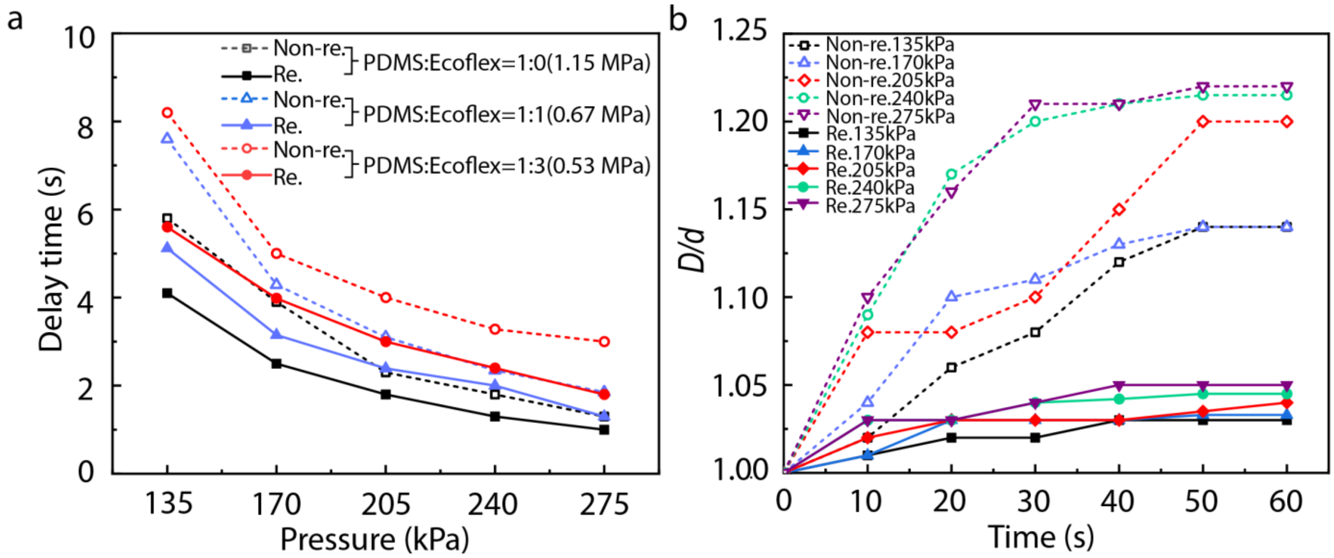

Supplementary Figure 5. Comparison of printing performance between reinforced and non-reinforced catheter robots. (a) Delay time measured for different moduli (variable weight ratio of PDMS184 to Ecoflex) of the robot under different applied pressures. The PDMS184 contained silicone elastomer base and curing agent in the weight ratio of 1:0.1, and the Ecoflex contains part A and part B in the weight ratio of 1:1. Both increasing the modulus of the robot body and manufacturing the robot body with a reinforcement layer significantly reduces the delay time improving the response of the soft catheter robot to the print commands while improving response performance. **(b)** Plot of expansion ratio *D/d* measured over time for both reinforced and non-reinforced catheters (*L*=100mm, *d*=1mm, catheter outer diameter *D*=4mm) under different applied pressures.

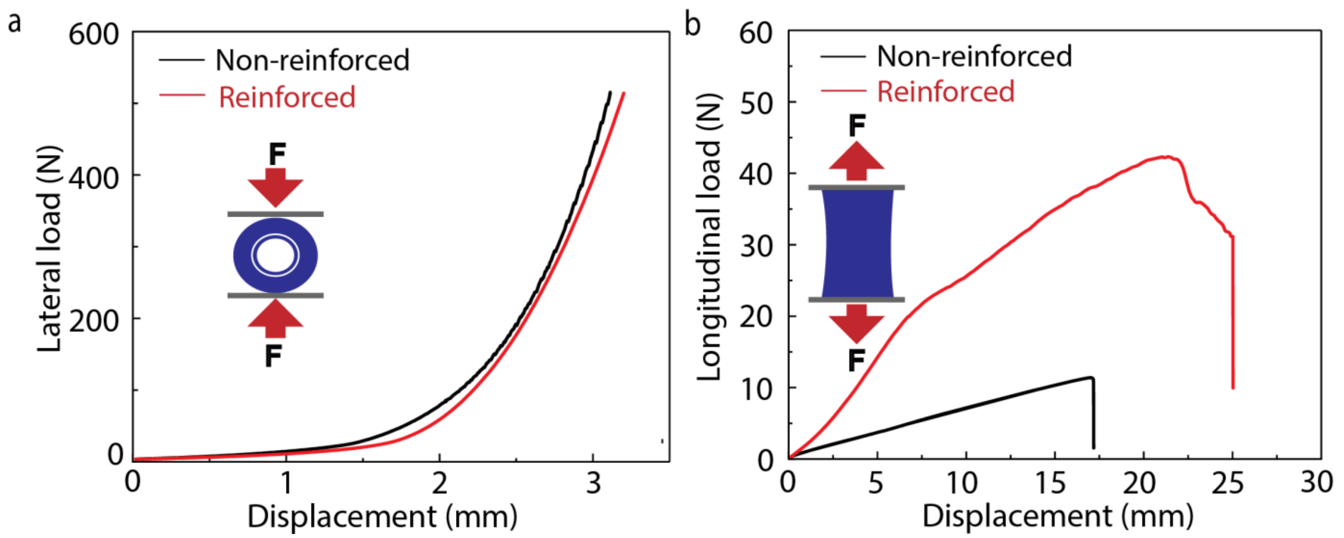

Supplementary Figure 6. Mechanical test of FSCR. (a) Diagram of lateral load of reinforced catheter robot and non-reinforced catheter robot specimens with the same diameter (Outer diameter is 4 mm, and inner diameter is 1 mm) and length (10 mm) versus displacement. **(b)** Diagram of longitudinal tensile strength measurement of the catheter robot with same diameter (Outer diameter was 4 mm, and inner was 1 mm) and test distance (60mm). The longitudinal tensile strength of the reinforced catheter robot and non-reinforced catheter robot are about 41.4 N and 10.8 N, respectively; the rupture strains for the reinforced catheter robot and non-reinforced catheter robot are about 250% and 180%, respectively.

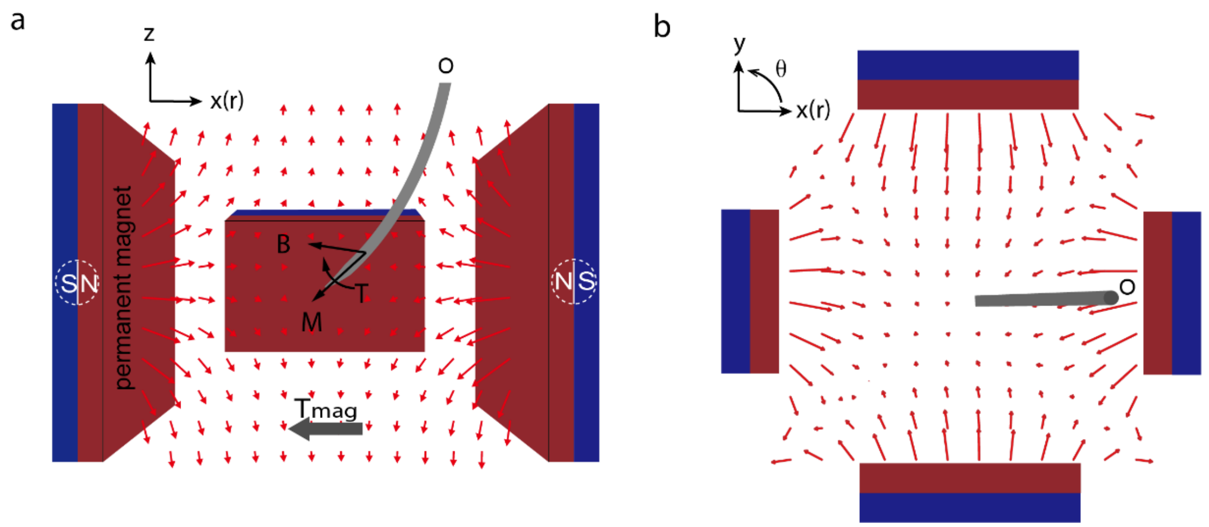

Supplementary Figure 7. Schematic of layout of four cuboidal magnets. (a) Equilibrium state of a ferromagnetic soft catheter robot under an applied magnetic field in a frontal view. **(b)** Top view of the equilibrium state of a ferromagnetic soft catheter robot under an applied magnetic field.

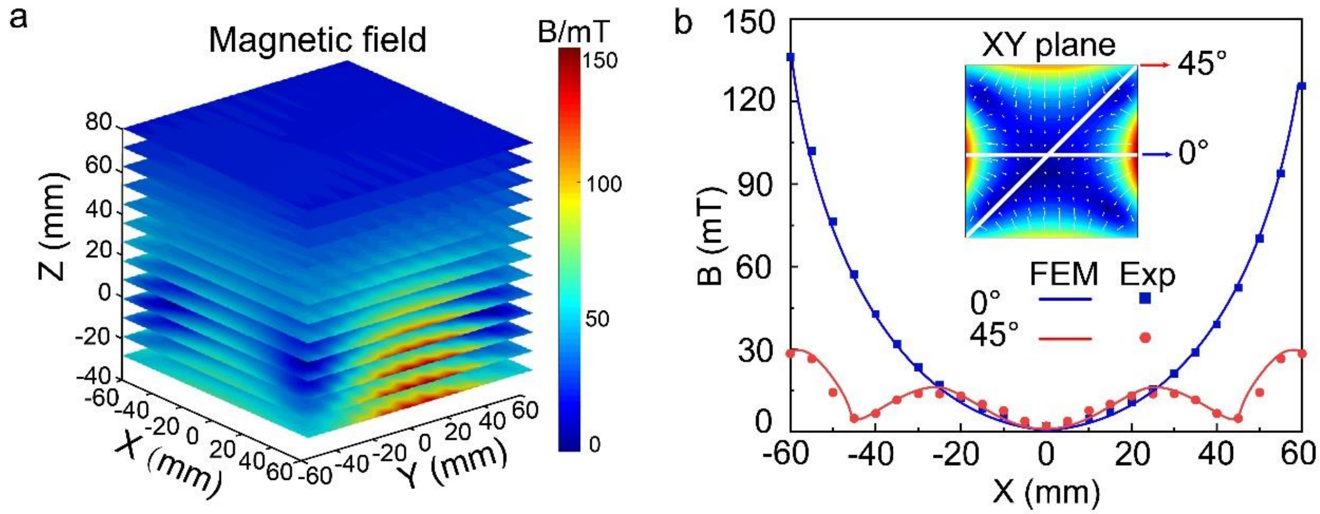

Supplementary Figure 8. Comparison between an analytical calculation and experimental measurements of the applied magnetic field distribution. (a) The measured three-dimensional distribution of the magnetic field. **(b)** Comparison of a finite element model (FEM) calculation and experimental measurements of the magnetic strength distribution in the XY plane at 0 °and 45 °relative to the X-axis. The background color represents the magnetic field strength and the arrows indicate the magnetic field vectors.

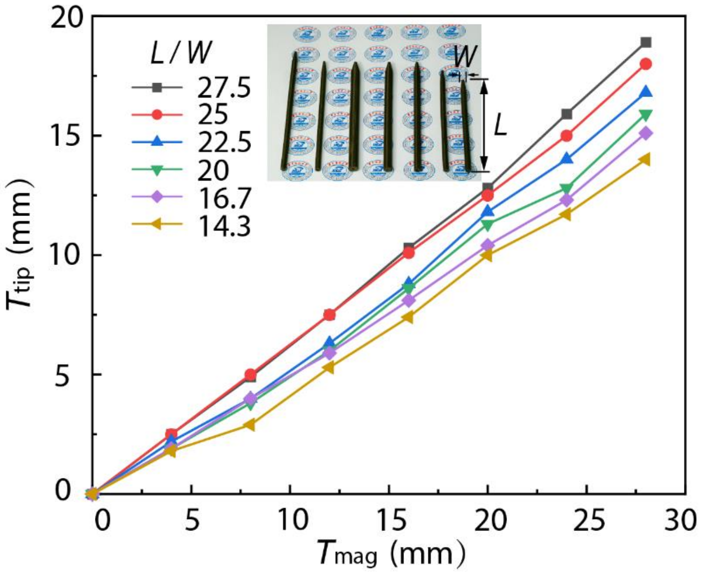

Supplementary Figure 9. Workspace for different aspect ratios of the FSCRs. Different equilibrium positions of the tip of the FSCR as the magnetic field is translated as a function of different robot body length (*L*) to diameter (*W*) ratios. As the *L/W* increases, the equilibrium position is further away from the center point thereby expanding the printing workspace.

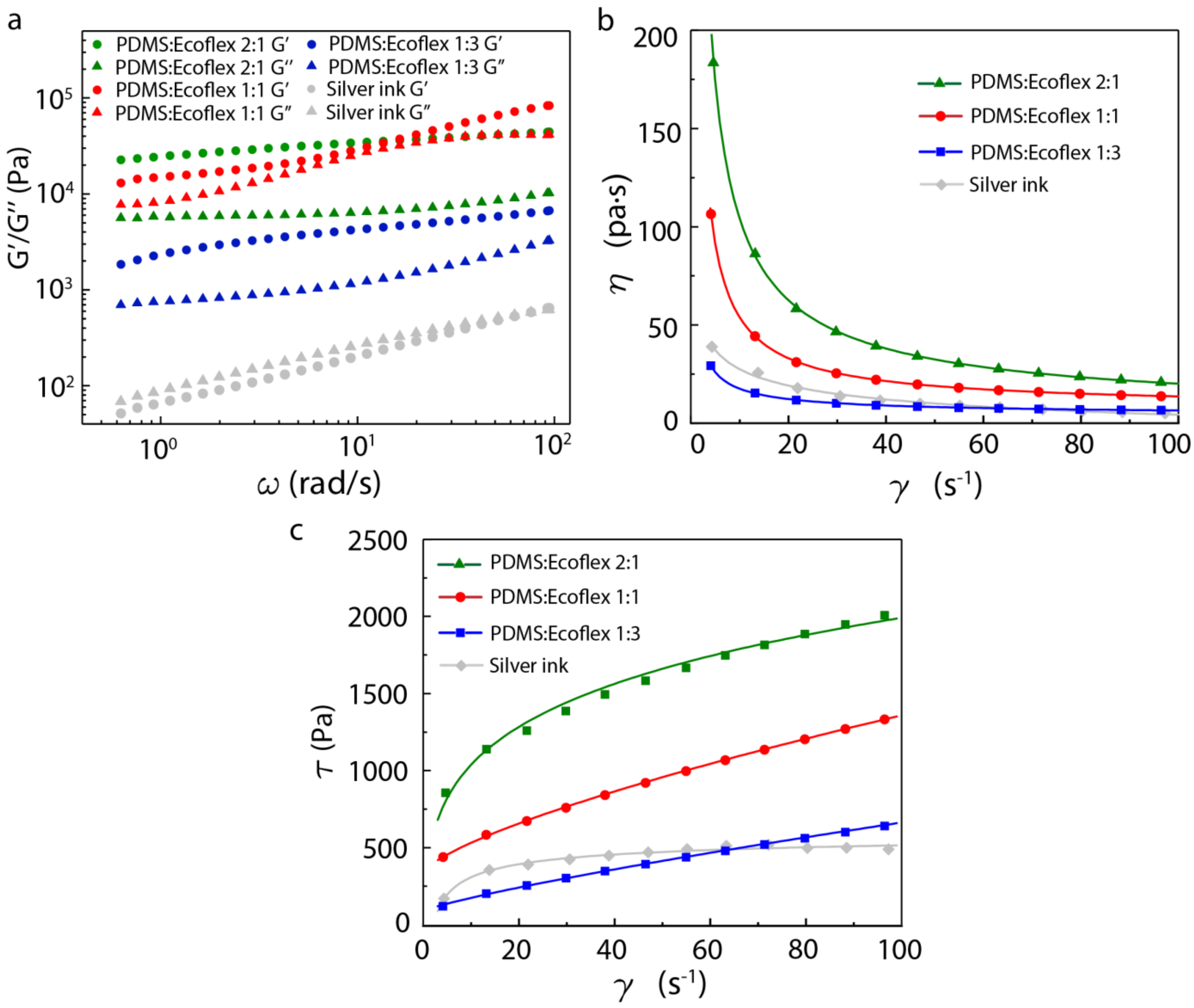

Supplementary Figure 10. Rheological properties of inks. (**a**) Logarithmic plot of the storage modulus G' and loss modulus G'' as a function of angular frequency ω. (**b**) Plot of the apparent viscosity η as a function of shear rate γ for viscoelastic inks. The zero-shear viscosity of printed inks are determined by a power law (or Ostwald Law) fit and yield about 339,248 mP · s, 581,935 mP · s , 71,170 mP · s and 66,980 mP · s for the mixture of PDMS-Ecoflex inks and the silver ink. **(c)** Plot of shear stress τ as a function of shear rate γ , where the curve of mixture of PDMS-Ecoflex was fitted by Herschel-Bulkley model while the curve of silver ink was fitted by a power law.

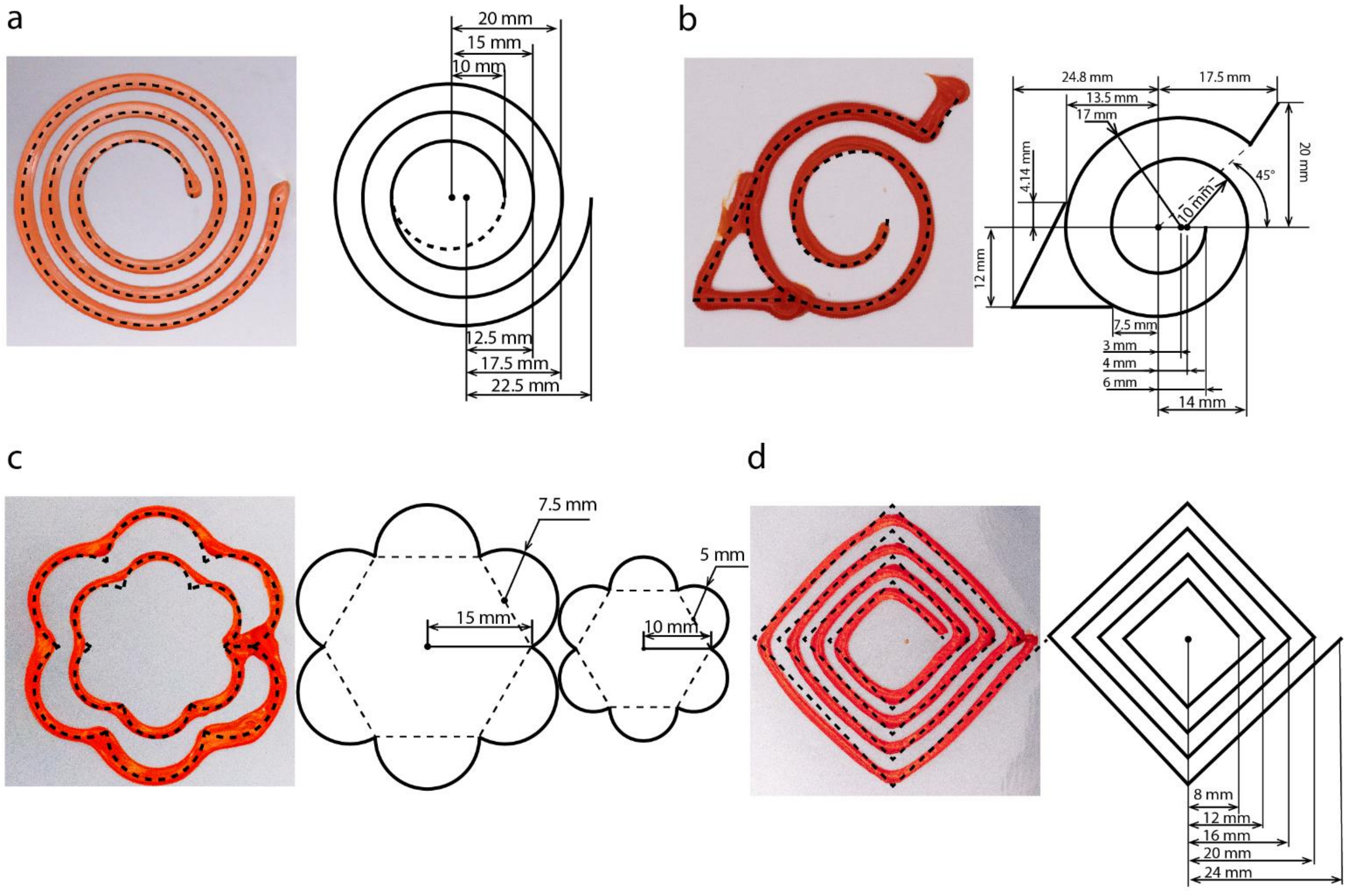

Supplementary Figure 11. Mode, designs and the faithfulness of printed pattern with which the pattern reproduces the model. (a) Spiral pattern. (b) Naruto symbol. (c) A flower with six petals. (d) Square spiral. In each panel, the printed pattern is on the left and the pattern design with parameters is on the right.

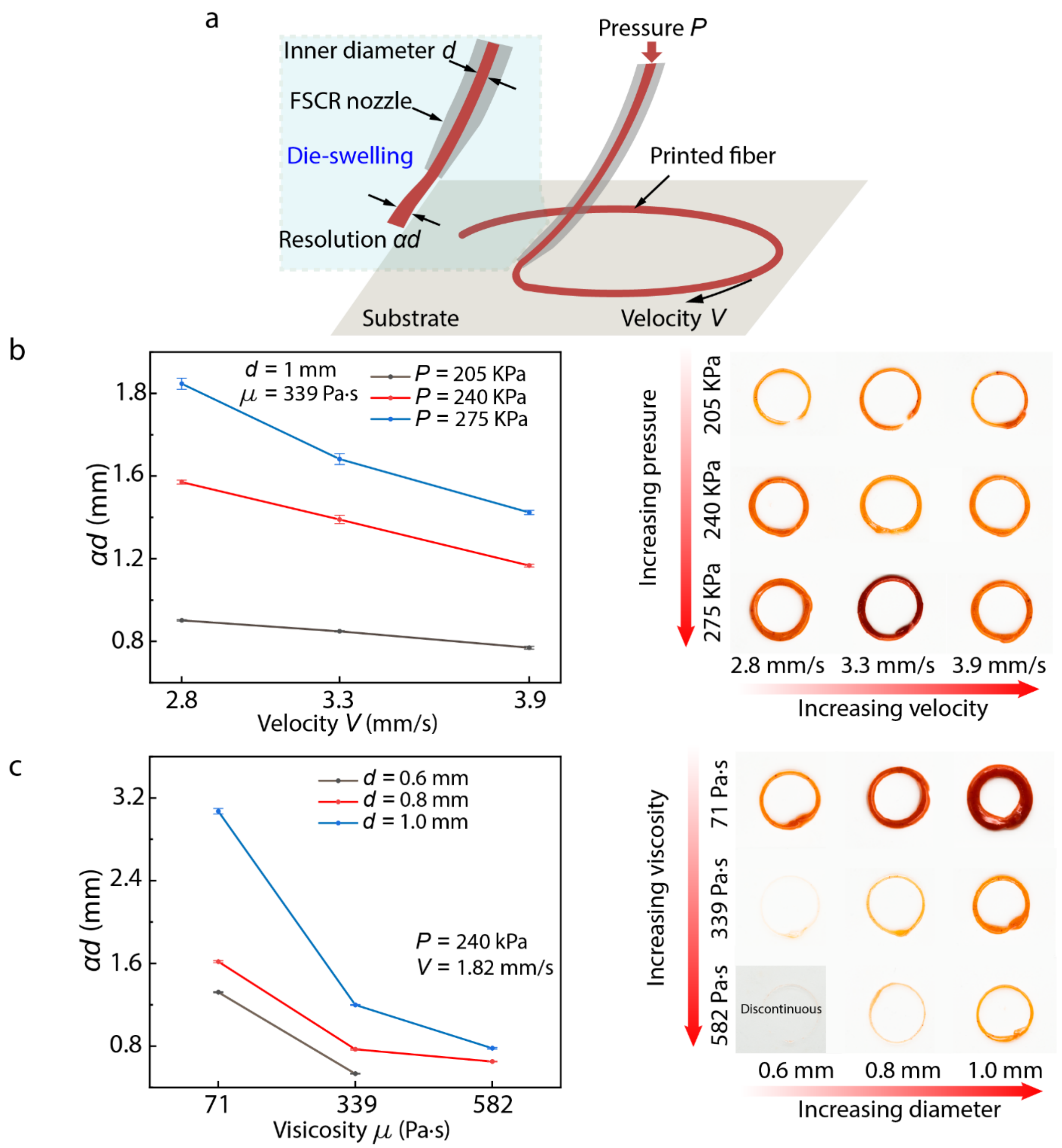

Supplementary Figure 12. **The resolution of printed fiber.** (a) Schematic illustration of die-swelling phenomenon after ink extrusion. The FSCR nozzle moves at a velocity of *V*, and the inks are extruded out of the nozzle at a pressure of *P*, with inner diameter *d*. The printing resolution is characterized by the diameter of the extruded fiber, denoted as of αd. (b) Increasing velocity *V* stretches the printed fiber thus reduces αd, while increasing the applied pressure P increases αd. (c) Increasing the size of inner diameter *d* of FSCR increases αd, while increasing the viscosity of printed fibers reduces αd. All the Error bars represent Standard Deviation (n=3).

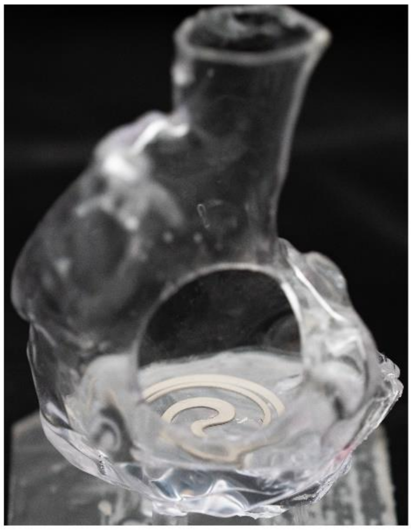

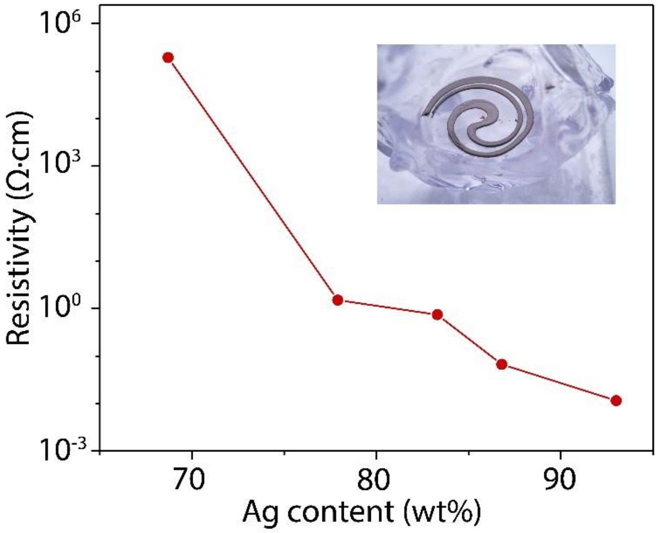

Supplementary Figure 13. The conductivity of the dried conductive ink as a function of silver content. The plot of square resistivity of the printing silver ink as a function of dried silver flake content (dried in 37℃ for 10 min).

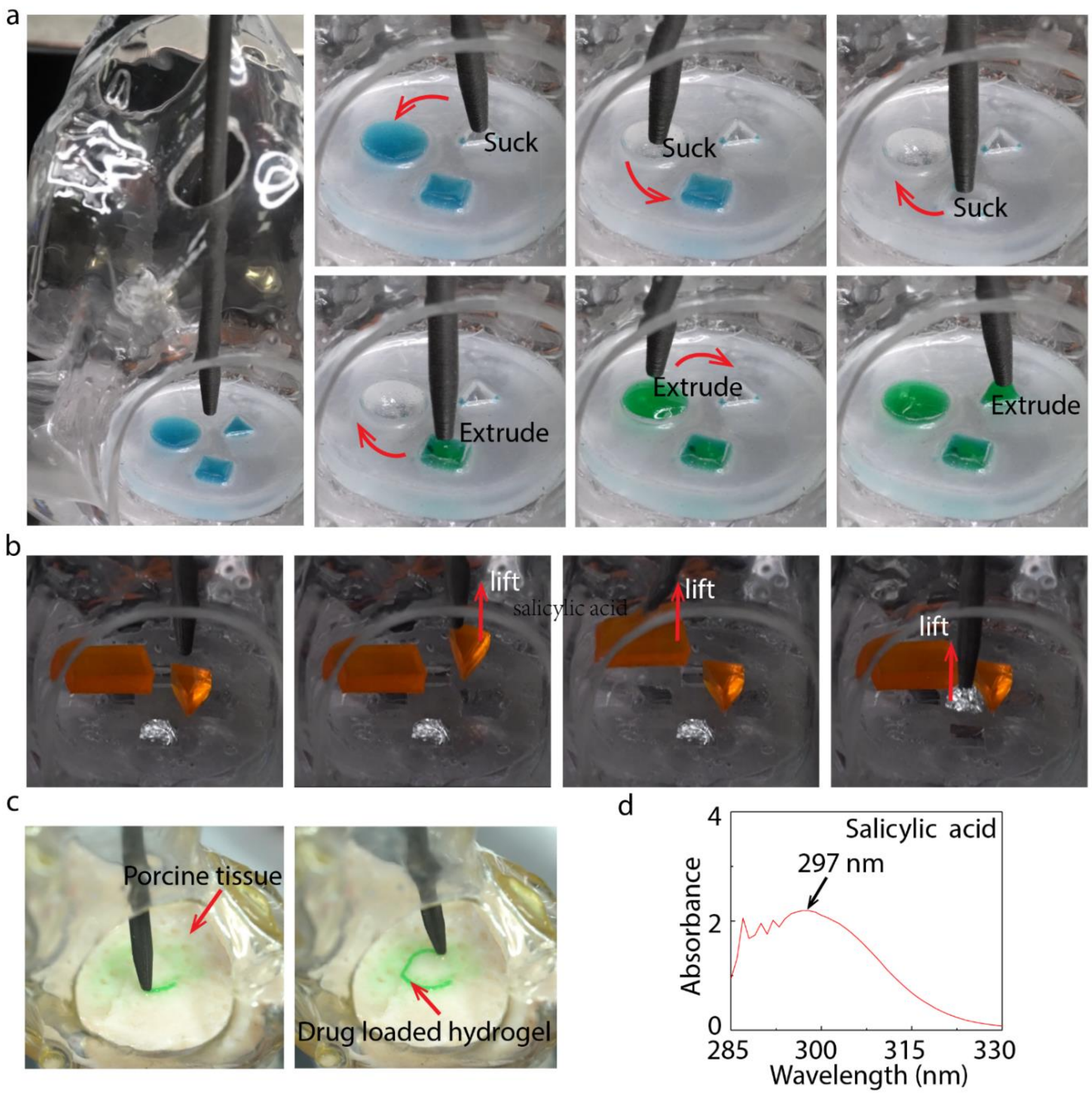

Supplementary Figure 14. Material delivery based on digital magnetic actuation. (a) Experimental demonstration of suction (4% sodium alginate solution dyed blue) and extrusion (4% sodium alginate solution dyed green) in a pre-defined pathway having different geometric sinks along the path. **(b)** The FSCR is able to lift the solid blocks with different geometric shapes (weight from 0.5g to 5g). The location of all the objects is random. **(c)** Printing dyed acetylsalicylic acid (ASA)-loaded hydrogel in a piece of porcine tissue which is immersed in PBS solution (pH 7.4). **(d)** Absorbance spectra of salicylic acid (SA). After sampling the mixing solution, the released drug is determined using a UV–vis spectrophotometer. ASA solutions undergo hydrolysis forming SA which intrinsic absorbance (peak height) is 297 nm.

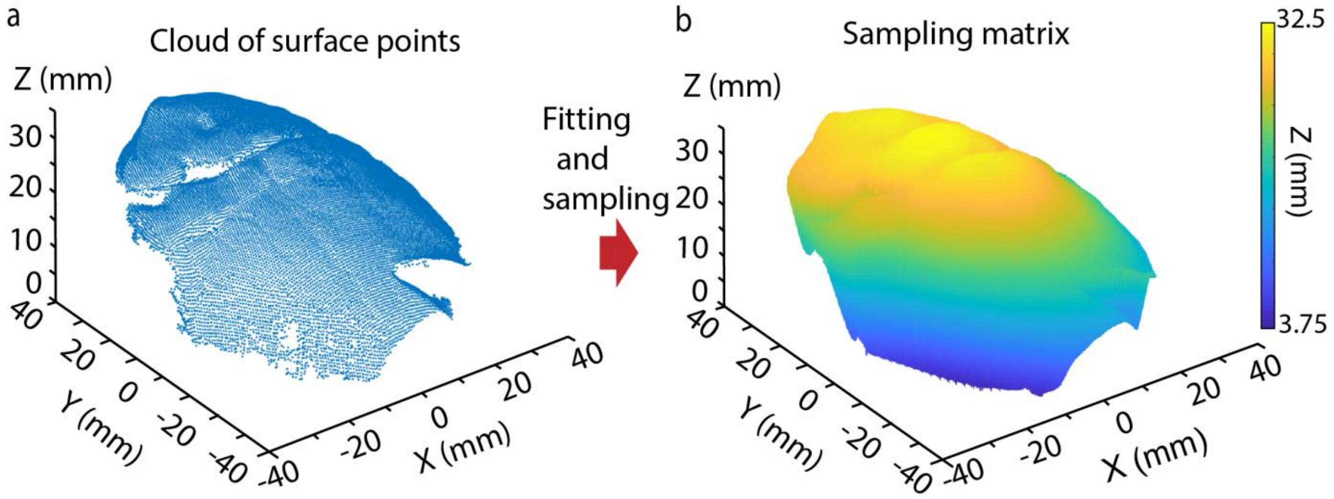

Supplementary Figure 15. Reconstruction of the curved surface. **(a)** Cloud of surface points from the 3D scan results of the porcine tissue surface. **(b)** The surface fitted from the cloud of surface points with the fit sampled at equal intervals of 0.1 mm; this data set is used to establish a surface parameter matrix. The color represents the height magnitude.

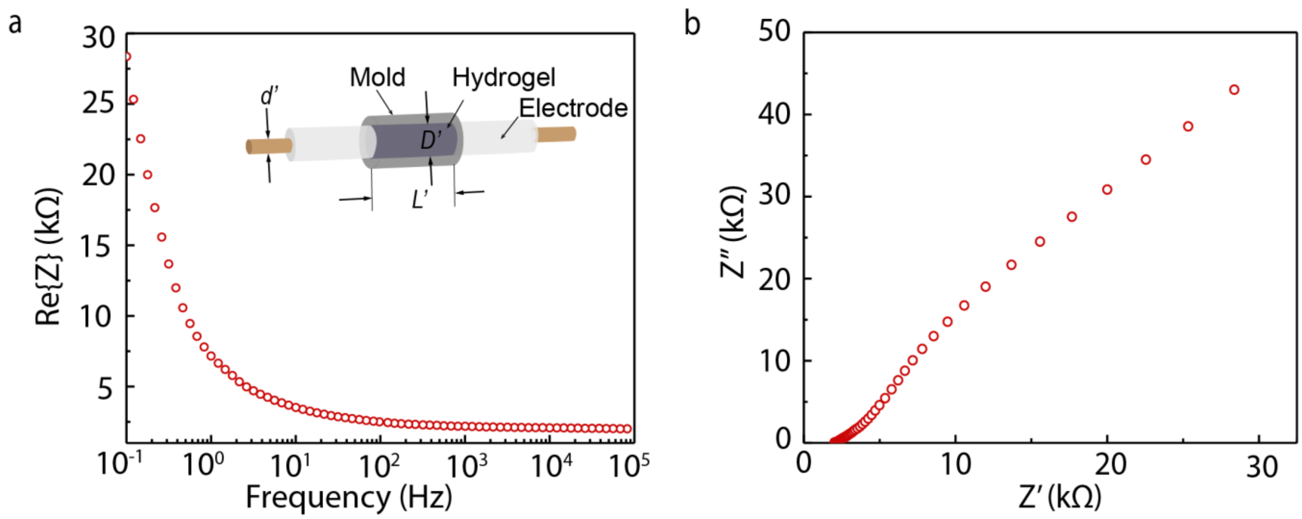

Supplementary Figure 16. Electrochemical impedance spectroscopy of a conducting hydrogel. **(a)** *Z* vs frequency curve of the conducting hydrogel (inset: illustration of two probe method to measure the impedance of the hydrogel). The gap *L'* between the two glass carbon electrodes, the inner diameter *D'* of cylindrical mold, and the diameter of electrode *d'* were 3 mm, 6 mm and 3 mm, respectively. **(b)** Nyquist plot of the conducting hydrogel.

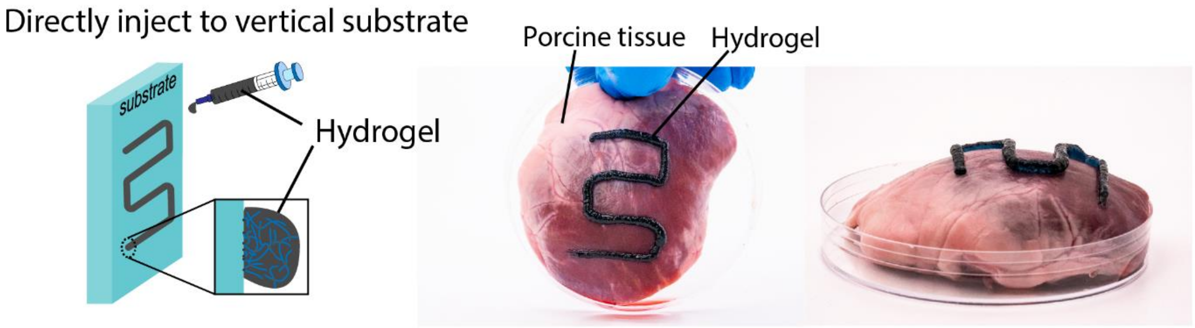

Supplementary Figure 17. Demonstration of adhesive properties of hydrogel for printing in situ. The hydrogel was directly injected to vertically placed porcine heart slice and it can maintain the shape well without falling.

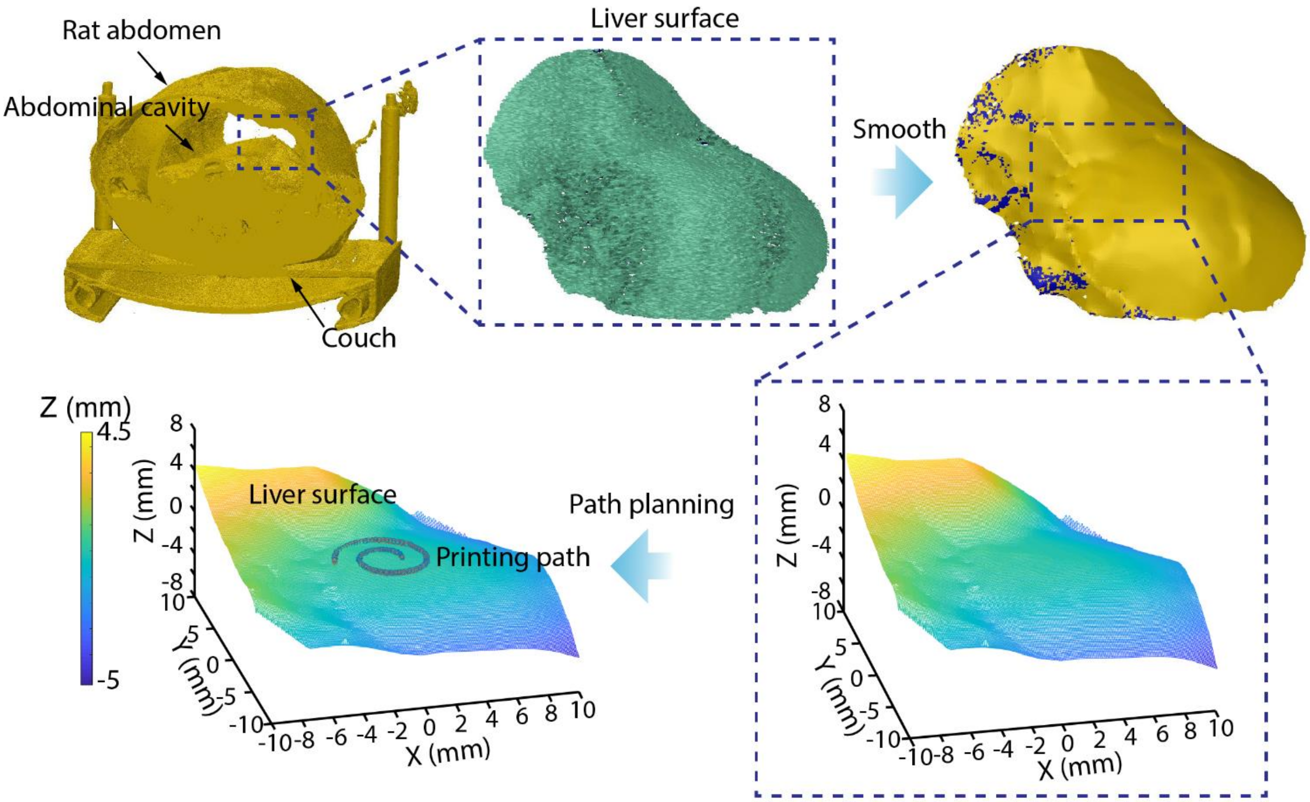

Supplementary Figure 18. Planning the printing path for minimally invasive bioprinting in vivo. Reconstruction of a 3D model based on CT of the liver and extraction of the smooth liver surface (top panels and inset). That surface is then sampled at equal intervals of 0.1 mm to establish a surface parameter matrix. Comparison of 3D surface matrix and printing path (lower left panel). The color represents the height magnitude.

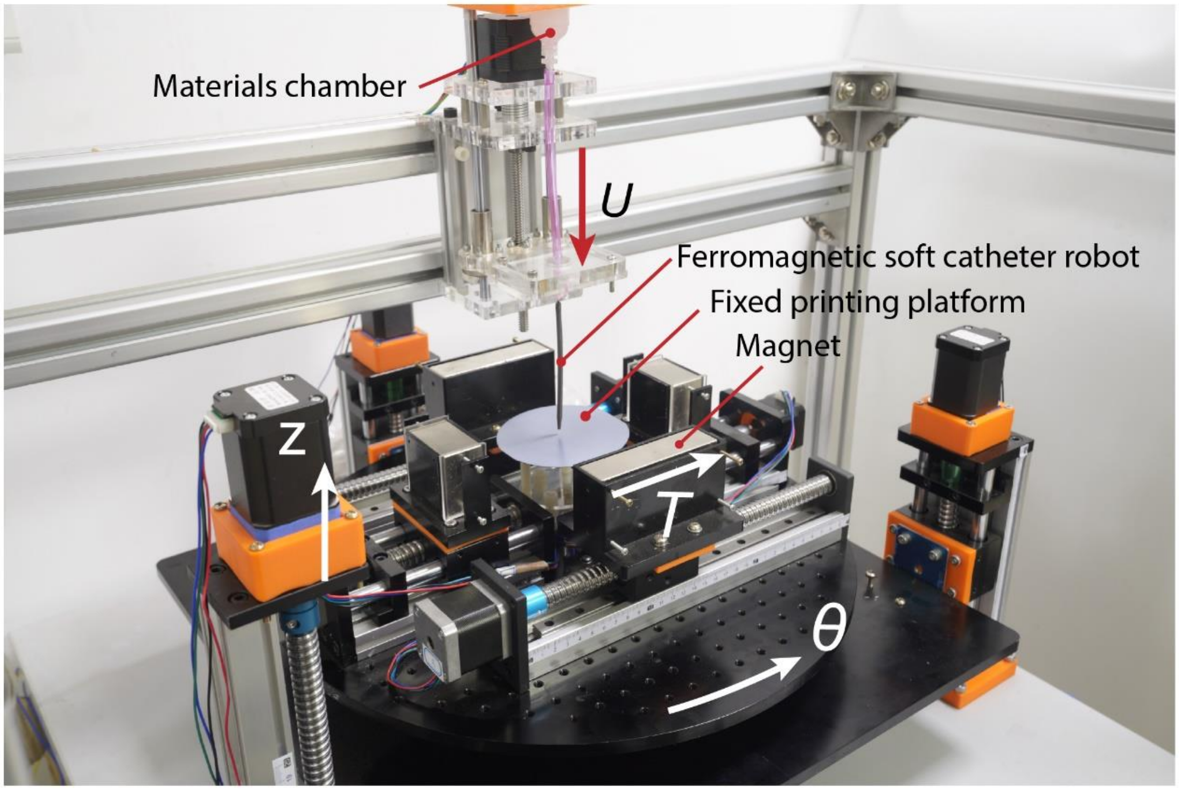

Supplementary Figure 19. Image of the physical apparatus for printing.
