# Supplementary material for: Ferromagnetic soft catheter robots for minimally invasive bioprinting
Source: Nat Commun. 2021 Aug 20;12:5072. doi: 10.1038/s41467-021-25386-w (PMC8379157; doi:10.1038/s41467-021-25386-w)
Supplement: Supplementary file 3 — Description of Additional Supplementary Files [file 41467_2021_25386_MOESM3_ESM.pdf]

Description of Additional Supplementary Files

Supplementary Movie 1. Comparison of expending during extrusion between non-reinforced and reinforced catheters. The extruded material (mixture of PDMS 1700 and Ecoflex part-A) of the two processes was the same.

Supplementary Movie 2. Demonstration of printing a flower with six petals pattern on planar platform by FSCR.

Supplementary Movie 3. Demonstration of printing a square spiral in the same setup.

Supplementary Movie 4. Demonstration of printing a nonunicursal animated symbol in the same setup.

Supplementary Movie 5. Demonstration of printing a fivelayered circular tube and scaffold in the same setup.

Supplementary Movie 6. Printing a wireless electronic device into a spiral pattern on the bottom of a chamber with conductive silver ink. The spiral conductive coil can relate to an electronic component such as a commercial light-emitting diode (LED), when actuated by an alternating magnetic field.

Supplementary Movie 7. Demonstration of numerically controlled material suction and extrusion of liquid to desired position. The suction and extrusion process were controlled by air pump, the mode of which is switched manually.

Supplementary Movie 8. Demonstration of numerically controlled lifting of solid buckles. Move the targeted materials with different shapes and variable weight (0.5 - 5 g) in confined environments.

Supplementary Movie 9. Demonstration of minimally invasive in vitro bioprinting on a natural surface of porcine tissue. The conducting hydrogel pattern matches the curved surface.

Supplementary Movie 10. Demonstration of minimally invasive in vivo bioprinting on a liver of rat model. The conducting hydrogel pattern matches the liver surface.
