# Supplementary material for: Ferromagnetic soft catheter robots for minimally invasive bioprinting
Source: Nat Commun. 2021 Aug 20;12:5072. doi: 10.1038/s41467-021-25386-w (PMC8379157; doi:10.1038/s41467-021-25386-w)
Supplement: Supplementary file 14 — Reporting Summary [file 41467_2021_25386_MOESM14_ESM.pdf]

nature portfolio

Corresponding author(s): Jianfeng Zang; Liu Wang

Last updated by author(s): Jul 6, 2021

Reporting Summary

Nature Portfolio wishes to improve the reproducibility of the work that we publish. This form provides structure for consistency and transparency in reporting. For further information on Nature Portfolio policies, see our Editorial Policies and the Editorial Policy Checklist.

Statistics

For all statistical analyses, confirm that the following items are present in the figure legend, table legend, main text, or Methods section.

n/a	Confirmed	
☐	☒	The exact sample size (*n*) for each experimental group/condition, given as a discrete number and unit of measurement
☐	☒	A statement on whether measurements were taken from distinct samples or whether the same sample was measured repeatedly
☒	☐	The statistical test(s) used AND whether they are one- or two-sided<br>*Only common tests should be described solely by name; describe more complex techniques in the Methods section.*
☒	☐	A description of all covariates tested
☐	☒	A description of any assumptions or corrections, such as tests of normality and adjustment for multiple comparisons
☐	☒	A full description of the statistical parameters including central tendency (e.g. means) or other basic estimates (e.g. regression coefficient) AND variation (e.g. standard deviation) or associated estimates of uncertainty (e.g. confidence intervals)
☒	☐	For null hypothesis testing, the test statistic (e.g. *F*, *t*, *r*) with confidence intervals, effect sizes, degrees of freedom and *P* value noted<br>*Give P values as exact values whenever suitable.*
☒	☐	For Bayesian analysis, information on the choice of priors and Markov chain Monte Carlo settings
☒	☐	For hierarchical and complex designs, identification of the appropriate level for tests and full reporting of outcomes
☒	☐	Estimates of effect sizes (e.g. Cohen's *d*, Pearson's *r*), indicating how they were calculated

Our web collection on statistics for biologists contains articles on many of the points above.

Software and code

Policy information about availability of computer code

Data collection	XProgram 4.3 (SCANTECH) was used for collecting scan data. The custom codes used in this study for minimally invasive bioprinting are available from github.
Data analysis	Matlab 2017a was used for analysis the data. Abaqus 2017 was used for simulation.

For manuscripts utilizing custom algorithms or software that are central to the research but not yet described in published literature, software must be made available to editors and reviewers. We strongly encourage code deposition in a community repository (e.g. GitHub). See the Nature Portfolio guidelines for submitting code & software for further information.

Data

Policy information about availability of data

All manuscripts must include a data availability statement. This statement should provide the following information, where applicable:

- Accession codes, unique identifiers, or web links for publicly available datasets
- A description of any restrictions on data availability
- For clinical datasets or third party data, please ensure that the statement adheres to our policy

All the code and data can be available in github link https://github.com/softnano501.

Field-specific reporting

Please select the one below that is the best fit for your research. If you are not sure, read the appropriate sections before making your selection.

- [x] Life sciences
- [ ] Behavioural & social sciences
- [ ] Ecological, evolutionary & environmental sciences

For a reference copy of the document with all sections, see nature.com/documents/nr-reporting-summary-flat.pdf

Life sciences study design

All studies must disclose on these points even when the disclosure is negative.

Sample size	For the in vitro experiments, at least 3 biological independent replicates were used, and 3 technical replicates of each were analysed.<br>For the in vivo experiments, at least 3 biological independent replicates were used, and 3 technical replicates of each were analysed.<br>For cytotoxic tests, sample size was selected for a minimum of n=3.<br>Minimally invasive bioprinting in vivo can only verify the effectiveness of this method. This is no sample size calculation.
Data exclusions	No data were excluded from the analysis.
Replication	The minimally invasive bioprinting experiments in vivo were confirmed by individual experiment at several hours interval. All the experiments were replicable but small adjustments. This was dependent to complex characteristics of printed materials such as sensitivity to environment, different rheological behaviours.
Randomization	Not applicable. Minimally invasive bioprinting in vivo can only verify the effectiveness of this method. There is no control group, and random method cannot be used.
Blinding	Not applicable. Minimally invasive bioprinting in vivo can only verify the effectiveness of this method. There is no control group, and blind method cannot be used.

Reporting for specific materials, systems and methods

We require information from authors about some types of materials, experimental systems and methods used in many studies. Here, indicate whether each material, system or method listed is relevant to your study. If you are not sure if a list item applies to your research, read the appropriate section before selecting a response.

Materials & experimental systems

n/a	Involved in the study	
x		Antibodies
	x	Eukaryotic cell lines
x		Palaeontology and archaeology
	x	Animals and other organisms
x		Human research participants
x		Clinical data
x		Dual use research of concern

Methods

n/a	Involved in the study	
x		ChIP-seq
x		Flow cytometry
x		MRI-based neuroimaging

Eukaryotic cell lines

Policy information about cell lines

Cell line source(s)	HCV-29 cell line, Human bladder epithelial cells, American Type Culture Collection.
Authentication	STR Authentication.
Mycoplasma contamination	No Mycoplasma contamination.
Commonly misidentified lines<br>(See ICLAC register)	None

Animals and other organisms

Policy information about studies involving animals; ARRIVE guidelines recommended for reporting animal research

Laboratory animals	We performed experiments in SD rat, 200-300g(male), 8-10 weeks.
Wild animals	No wild animals were used in this study.

Field-collected samples	No field-collected samples were used in this study.
Ethics oversight	All the animal experiments were approved by the Animal Care and Use Committee of Tongji Hospital, Tongji Medical College, Huazhong University of Science and Technology, Hubei, China.

Note that full information on the approval of the study protocol must also be provided in the manuscript.
